# Sublethal effects of bifenazate on biological traits and enzymatic properties in the *Panonychus citri* (Acari: Tetranychidae)

**DOI:** 10.1038/s41598-021-99935-0

**Published:** 2021-10-22

**Authors:** Hongyan Wang, Tianrong Xin, Jing Wang, Zhiwen Zou, Ling Zhong, Bin Xia

**Affiliations:** 1 School of Life Sciences, Nanchang University, Nanchang, 330031 People’s Republic of China; 2 Development & Service Center for Agriculture and Rural Industry of Jiangxi Province, Nanchang, 330096 People’s Republic of China

**Keywords:** Ecology, Evolution

## Abstract

*Panonychus citri*, a major citrus pest. In pest management, bifenazate is a novel acaricide with high biological activity against red mites, such as *Tetranychus urticae Koch*. However, in the field, pests are frequently exposed to sublethal or lethal concentrations of pesticides. At present, its sublethal effects on *P. citri* have not been reported. Therefore, in order to investigate sublethal effect of bifenazate on biological traits and enzymatic properties of *P. citri*. The newly emerged females were treated with two concentrations of bifenazate: LC_10_ and LC_30_, the development and fecundity were observed. The results showed that female adult duration, fecundity, oviposition days, longevity were decrease compared with control, but pre-oviposition period was longer, net reproductive rate (*R*_0_), mean generation (*T*) were decreased, intrinsic rate of increase (*rm*), finite rate (*λ*) were decreased in LC_30_, however, doubling time was increased. Enzymatic tests showed that CAT, POD, CarE activities were higher in treatments than control. The SOD and GST activities were lower in LC_30_ than control and LC_10_, the CYP450 activity was decreased with the increasing concentrations. This study demonstrated that low lethal concentrations of bifenazate adversely affected life table parameters, enzymatic properties in *P. citri*. Therefore, bifenazate has the potential to control this pest.

## Introduction

*Panonychus citri* (Acarina: Tetranychidae) is a spider mite with worldwide distribution^1^. The *P. citri* has a stabbing mouthpiece and feeds mainly on the leaves of Citru^2^. Currently, the main strategy for *P. citri* control remains chemical control, however, the continued application of chemical insecticides as the favored method for controlling *P. citri*, has resulted in widespread insecticide resistance in this pest, as well as being responsible for reductions in the populations of natural enemiesit^3–6^, Therefore, it is necessary to select more effective acaricides for controlling this pest.

Bifenazate is a novel acaricide developed in recent years, which is toxic to leaf mites at all life stages^7^. At present, it is used to control spider mites on a variety of crops, including fruits and ornamental plants^8^. Additional, bifenazate has low toxicity to mammals and aquatic organisms. It has rapid knockdown and no cross resistance with other acaricides. These properties make bifenazate an ideal insecticide for spider mites control^7^.

The impact of pesticides on insects is reflected not only in killing pests directly but also in the effects of insect populations by affecting the fecundity, insect longevity and physiological traits^9–11^. After one application in the field, the concentrations of pesticides may be different in diverse parts of the field, so insect populations are exposed to different concentrations of pesticides. Different concentrations of pesticide have various results on the physiology, biochemistry and biological characteristics of insects^12^. Traditionally, measurement of the acute toxicity of pesticides to arthropods has relied largely on the determination of an acute median lethal dose or concentration. However, the estimated lethal dose during acute toxicity tests may only be a partial measure of the deleterious effects^13–15^. In certain cases, sublethal concentration of insecticides can reduce insects’ survival or fecundity, whereas in other cases, sublethal concentration of insecticides can stimulate growth and reproduction among insects, producing hormetic effects^16^. For example, cyantraniliprole at a low lethal concentration (LC_30_) significantly inhibited fecundity in *Helicoverpa Assa*^17–19^. However, (*T. urticae*) fecundity has been stimulated after exposure to spinetoram at sublethal and low lethal concentrations (LC_10_ and LC_20_)^20^. Meanwhile, in present study, six key enzymes of detoxification metabolism were selected to determine enzyme activity. The main enzymes involved in the phase I and phase II detoxification processes are P450 monooxygenase, glutathione S-transferase (GST), and carboxylesterase (CarE)^21^. Superoxide dismutase (SOD), peroxidase (POD), and catalase (CAT) are three important protective enzymes in insects that play roles in immunity, preventing free-radical-associated damage, and protecting cells from adverse environmental effects^22^. In some cases, the low lethal concentration of insecticides can inhibit the enzyme activity in insects, while in other cases, the low lethal concentration of insecticides can stimulate the enzyme activity. For example, it has been reported that low lethal concentrations (LC_10_ and LC_25_) of abamectin can promote upregulation of the SOD, POD, and CAT activities in *Diadegma semiclausum*, with activity increasing with increasing insecticide concentration^23^ In contrast, the levels of SOD, POD, and CAT activity in *Harmonia axyridis* were shown to decrease with an increase in abamectin concentration^24^. Avermectin (LC_10_ and LC_25_) resulted in a significant induction of CarE, GST activities in *Sogatella furcifer*^25^. Previously, it was found that GST and P450 activities in *Aphis craccivora* were significantly induced after treatment with cycloxaprid and imidacloprid (LC_50_), whereas in contrast, the activity of the CarE activity was inhibited. Traditional estimating only by measuring the lethal effect of insecticides may underestimate the total effects of insecticides on the pest^26, 27^. Accordingly, in this study, we sought to gain insights into the roles of these enzymes to insecticide-induced stress. To this end, we exposed this insect to low lethal concentrations of bifenazate and subsequently monitored the changes in enzyme activity levels and biological traits. In pest management (IPM), it has a significant influence on the sublethal effects of insecticides.

The objective of present study was to obtain a comprehensive understanding of the sublethal effects of bifenazate on *P. citri*, including its developmental time, fecundity, life table parameters and detoxification enzymes, antioxidant enzyme^28^. The results may be employed to understand the sublethal effects of bifenazate on *P. citri*, which will contribute to the assessment and rational application of bifenazate for controlling this pest. Overall, research on the effects of acaricides on *P. citri* populations will be invaluable in evaluating the role that particular acaricides play in sustainable management programs involving the pest mite. Moreover, knowledge on the activity of detoxification enzymes in *P. citri* may provide a basis for abating or minimizing the development of resistance to effective acaricides^29^.

## Materials and methods

### Mite and pesticide

The laboratory strains of *P. citri* were collected from the citrus orchard in Wuning County, Jiujiang City, Jiangxi Province, China, In September 2019. The population was reared on the leaf disc of *Aurantii Fructus* in climate-controlled chamber, under the conditions of 26 ± 1 °C, relative humidity 70% ± 10%, photoperiod 16 h: 8 h (L:D). Place on a wet sponge in a Petri dish (15 cm in diameter), cotton slivers were placed around each leaf to prevent mites from escaping, without exposure to insecticide. Bifenazate (C17H20N2O3; ≥ 99% Purity) was provided by accustandard (New Haven, USA). All studies involving plants (*Fructus Aurantii* leaves) have been carried out in accordance with relevant institutional, national or international guidelines.

### Bioassay

Bioassay of female *P. citri* was performed according to the modified leaf dish dipping method of Ken and Yamamoto^30^. The newly emerged third instar female adult mites were transferred to the leaves of *Fr. aurantii* by small brush. 45 individuals were selected from each leaf. Wet cotton was put around the leaves with mites to prevent mites from escaping. The mites were placed in climate-controlled chamber. After 4 h, the mites were examined by microscope, the dead and inactive individuals were removed. Then, use tweezers to immerse the leaves with mites in different concentrations of acaricide solution for 5 s, and take out the excess liquid and quickly suck it up with absorbent paper. Leaves with female adult mites soaked in 0.1% ethyl acetate solution were set as control treatment. Put the treated leaves into the prepared leaf dish and put them into the climate control chamber. Each treatment was repeated in 4 groups. After 24 h, the mortality was recorded under microscope. If their foot does not move, it will be regarded as death. If the mortality of control group is less than 10%, it will be regarded as effective experiment.

### Low lethal concentration of bifenazate effects on the life-history traits of *P. citri*

**Figure 1:**
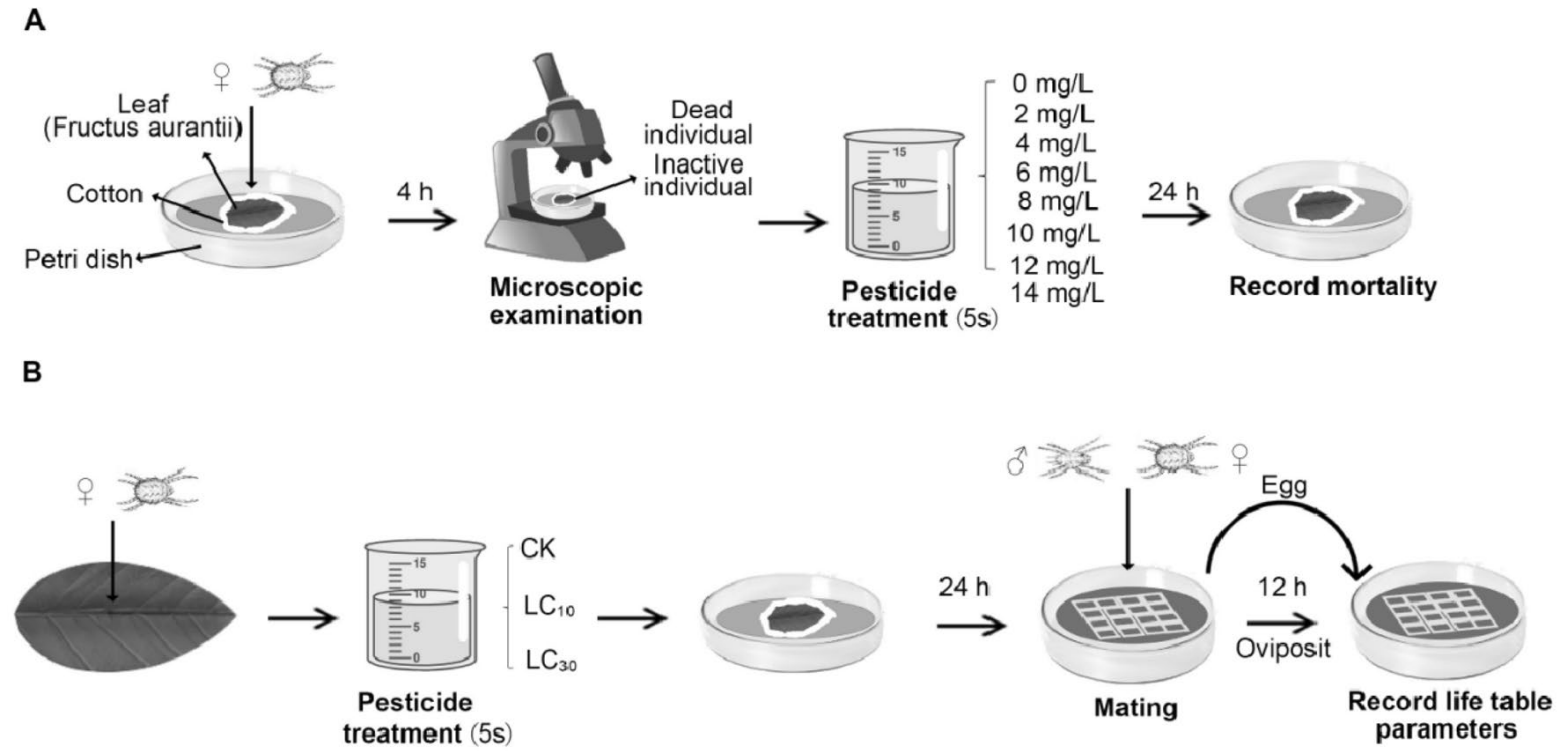
(**A**) Schematic diagram of bioassay method for bifenazate solution with eight concentrations. (**B**) Life table observation of female adults of *Panonychus citri* in three treatments (CK, LC_10_, LC_30_). Figure is drawn by Hongyan Wang with Photoshop2020 (https://www.adobe.com/products/photoshop.html).

In this study, leaf dipping method and leaf dish feeding method were used, and the feeding table was made as shown in the Fig. 1. The third instar female adult mites were selected and placed on each leaf dish. After 4 h, the leaves and female adult mites were soaked with bifenazate (LC_10_, LC_30_) by leaf dish dipping method. The leaves with female adult mites were soaked with 0.1% ethyl acetate solution as control. Then they were raised in climate-controlled chamber. After 24 h, the survivors were selected to the fresh rearing platform to continue feeding, and the same number of male adult mites were selected at the same time. After mating for 12 h, 100 eggs were taken from each treatment for single feeding. When female mites are mature, male mites are selected for mating, and the fertility and mortality are observed every 24 h until all female adults die.

### Preparation of samples for enzyme assay

The female adult mites (as described in “Low lethal concentration of bifenazate effects on the life-history traits of *P. citri*” section) were exposure to bifenazate for 24 h at concentrations of LC_10_, LC_30_, and the survivors were picked up. 150 individuals were placed in a 1.5 mL centrifuge tube. The collected samples were treated with liquid nitrogen and stored at − 80 °C. There were four treatments in the experiment, and three replicates were set for each treatment.

### Measurement of tissue total protein

The purchased total protein extraction kit (Nanjing Jiancheng Bioengineering Institute, Nanjing, China) was used to determine the total protein content of the sample. PBS buffer (0.05 mol/L, pH 7) was added into the centrifuge tube containing 150 mites. After full homogenization, the homogenate was centrifuged at 4 ℃ and 14,000 rpm for 15 min, and then the supernatant was taken as the enzyme solution to be tested. The sample was added according to the sample adding system in the kit instructions. The reading was performed at 562 nm with Perkin Elmer.

### Measurement of antioxidant enzymes activities

#### Catalase (CAT)

According to the instruction of CAT test kit (Nanjing Jiancheng Bioengineering Institute, Nanjing, China), the activity of CAT was determined by ammonium molybdate method. Absorb 200 μL of reaction mixture and add it into 96 well plate. Read the OD value at 405 nm, repeat 3 times, and take the average value. The activity of CAT was calculated.

#### Superoxide dismutase (SOD)

Refer to the instruction of SOD activity test kit (Nanjing Jiancheng Bioengineering Institute, Nanjing, China). Mix the solution well, place it at room temperature for 10 min, absorb 200 μL accurately, and add the reaction solution into 96 well plate. At the wavelength of 550 nm, read the OD value of absorbance, repeat for 3 times, and take the mean value. The activity of SOD was calculated.

#### Peroxidase (POD)

The activity of POD was determined according to the instruction of POD assay kit (Nanjing Jiancheng Bioengineering Institute, Nanjing, China). After the solution was mixed, centrifuged at 3500 rpm for 10 min, 200 μL supernatant was added into 96 well plate. Read the OD value at the wavelength of 420 nm, repeat three times, and take the mean value. The activity of pod was calculated.

### Measurement of detoxifying enzyme activities

#### Carboxyl esterase (CarE)

According to the instructions of CarE assay kit (Nanjing Jiancheng Bioengineering Institute, Nanjing, China), the activity of CarE was determined by spectrophotometry. Fully mix the solution, take 5 μL supernatant and 1000 μL preheating working solution into 1 mL glass cuvette in turn, quickly mix them, and read the absorbance value after 10 s and 190 s at 450 nm. The change of absorbance value is the measured value, repeat for 3 times, and take the average value. Calculate the vitality of CarE.

#### Glutathione S-transferase (GSH-ST)

The activity of GSH-ST was determined according to the instruction of GSH-ST assay kit (Nanjing Jiancheng Bioengineering Institute, Nanjing, China). Mix the solution well, place it at room temperature for 15 min, absorb 200 μL reaction solution and add it into 96 well plate. Reading the OD value of absorbance at 412 nm, repeat 3 times, and take the average value. The activity of GSH-ST was calculated.

#### Cytochrome P450 (CYP450)

According to the instructions of insect cytochrome P450 Elisa kit (Nanjing Jiancheng Bioengineering Institute, Nanjing, China), the content of cytochrome P450 was detected by competitive method. Read the OD value of absorbance at the wavelength of 450 nm, repeat for 3 times, and take the average value. The determination should be carried out within 10 min after the termination of the solution. According to the concentration and OD value, the standard curve equation was calculated to calculate the activity of CYP450.

### Statistical analysis

Analyzed by probit analysis, using the SPSS program. The life-history raw data of all individuals were analyzed according to the age-stage and two-sex life table model as proposed Liu and Chi^31, 32^. The life table parameters were calculated accordingly, including age-stage specific survival rate (*s*_*xj*_) (where x = age in days and *j* = stage), age-specific survival rate (*l*_*x*_), age-specific fecundity (*m*_*x*_), adult preoviposition period (APOP, the length of the preoviposition period beginning from adult emergence), and total preoviposition period (TPOP, the length of the preoviposition period beginning from birth). Population growth parameters including net reproductive rate (*R*_*0*_), intrinsic rate of increase (*rm*), finite rate of increase (*λ*), and mean generation time (*T*) were also calculated as follows:

1. Net reproductive rate (*R*_*0*_): *R*_*0*_ = Σ*l*_*x*_*m*_*x*_;
2. Intrinsic rate of increase (*rm*): Σ*e*-*rm*(x + 1) *l*_*x*_*m*_*x*_ = 1
3. Mean generation time (*T*): *T* = ln *R*_*0*_/*rm*;
4. Finite rate of increase (*λ*): *λ* = *erm*.

The standard errors of the developmental times, survival, fecundity, longevity, and population parameters were estimated by using the bootstrap method with 100,000× resamplings^33^, the paired bootstrap test was used to compare differences^34^. The computer program TWOSEXMSChart was used to analyze the raw data and calculate population parameters. Both bootstrap and paired bootstrap test routines were included in this program.

## Results

### Toxicity of bifenazate to the third instar female adults of *P. citri*

The toxicity of bifenazate to the third instar female adults of *P. citri* was stronger (Table 1). The median lethal concentration (LC_50_) was 11.915 mg/L. the concentrations leading to 10% and 30% mortality were 3.625 mg/L and 7.015 mg/L, respectively.

**Table1:** Toxicity of bifenazate on female adults of *Panonychus citri*.

Insecticides	Concentration mg/L (95% CL) − 1			LC-P equation	χ^2^	R
	LC_10_ mg/L	LC_30_ mg/L	LC_50_ mg/L			
Bifenazate	3.63 (0.89–5.33)	7.02 (3.68–9.51)	11.96 (8.59–16.35)	y = − 2.453 + 2.279x	19.22	0.91

### Low lethal concentration of bifenazate effects on the life-history traits of *P. citri*

Through bioassay of the third instar female adults of *P. citri*, we obtained low and median lethal concentrations. Tables 2 and 3 show the effects on the life table parameters of F_1_ offspring of *P. citri*. The results showed that compared with the control group, there was no significant difference in the average immature development duration of female adults of *P. citri* treated with LC_10_ and LC_30_ (Table 2) Compared with adult pre-oviposition (APOP) and total pre-oviposition (TPOP) of control group, pre-oviposition and total pre-oviposition of LC_10_ and LC_30_ treatment were significantly prolonged. However, compared with the control, the maturity (13.011 days), longevity (23.194 days) and fecundity (5.178 eggs/female) were higher, LC_10_ and LC_30_ treatment significantly reduced maturity, longevity and fecundity (9.011 days, 19.706 days, 4.309 eggs/female and 7.744 days, 18.789 days, 3.413 eggs/female).

**Table 2:** Life tables of *Panonychus citri* exposed to low lethal concentrations of bifenazate.

Stage	Control	Bifenazate LC_10_	Bifenazate LC_30_
	Mean ± SE	Mean ± SE	Mean ± SE
Egg duration (days)	(4.90 ± 0.050)a	(4.98 ± 0.05)a	(5.06 ± 0.05)a
Larve duration (days)	(1.35 ± 0.044)a	(1.43 ± 0.047)a	(1.41 ± 0.06)a
Nymph duration (days)	(2.44 ± 0.047)a	(2.50 ± 0.045)a	(2.52 ± 0.04)a
Female adults duration (days)	(13.01 ± 0.08)c	(9.01 ± 0.07)b	(7.74 ± 0.07)a
Longevity (days)	(23.20 ± 0.09)c	(19.70 ± 0.09)b	(18.80 ± 0.11)a
APOP (days)	(1.49 ± 0.04)a	(1.79 ± 0.04)b	(2.07 ± 0.04)c
TPOP (days)	(10.18 ± 0.07)c	(10.69 ± 0.08)b	(11.05 ± 0.09)a
Oviposition (days)	(8.44 ± 0.06)c	(5.86 ± 0.10)b	(4.62 ± 0.073)a
Fecundity (eggs/female adult/day)	(5.18 ± 0.04)c	(4.31 ± 0.03)b	(3.41 ± 0.03)a

*APOP* adult pre-ovipositional period, *TPOP* total pre-ovipositional period (from egg to first oviposition).

Means in a row followed by the same letter are not significantly different by the boostrap paired test (*p* > 0.05).

**Table 3:** The sublethal effects of bifenazate on *Panonychus citri* population parameters.

Population parameters	Control	Bifenazate LC_10_	Bifenazate LC_30_
	Mean ± SE	Mean ± SE	Mean ± SE
Net reproductive rate (R_0_) (day^−1^)	(55.85 ± 1.98)a	(34.82 ± 1.83)b	(21.29 ± 0.70)c
Mean generation time (T) (day)	(15.28 ± 0.18)a	(14.38 ± 0.14)b	(13.73 ± 0.09)c
Intrinsic rate of increase (rm) (day^−1^)	(0.26 ± 0.01)a	(0.25 ± 0.01)a	(0.23 ± 0.01)b
Finite rate of increase (λ) (day^−1^)	(1.30 ± 0.01)a	(1.28 ± 0.01)a	(1.25 ± 0.02)b
Doubling time	(2.64 ± 0.05)b	(2.81 ± 0.07)b	(3.12 ± 0.03)a

Means in a row followed by the same letter are not significantly different by the boostrap paired test (*p* > 0.05).

Compared with the control, the net reproductive rate (R_0_) and average generation time (*T*) of female adult mites treated with LC_10_ and LC_30_ were significantly decreased, while the intrinsic rate of increase (*rm)* of insects treated with LC_10_ had no significant difference; however, The intrinsic rate of increase (*rm*), finite rate of increase (λ) for the LC_30_ treated group significantly decreased for the control group (Table 3); the population doubling time (DT) of LC_30_ group were prolonged, but there was no significant difference between LC_10_ group and control group.

**Figure 2:**
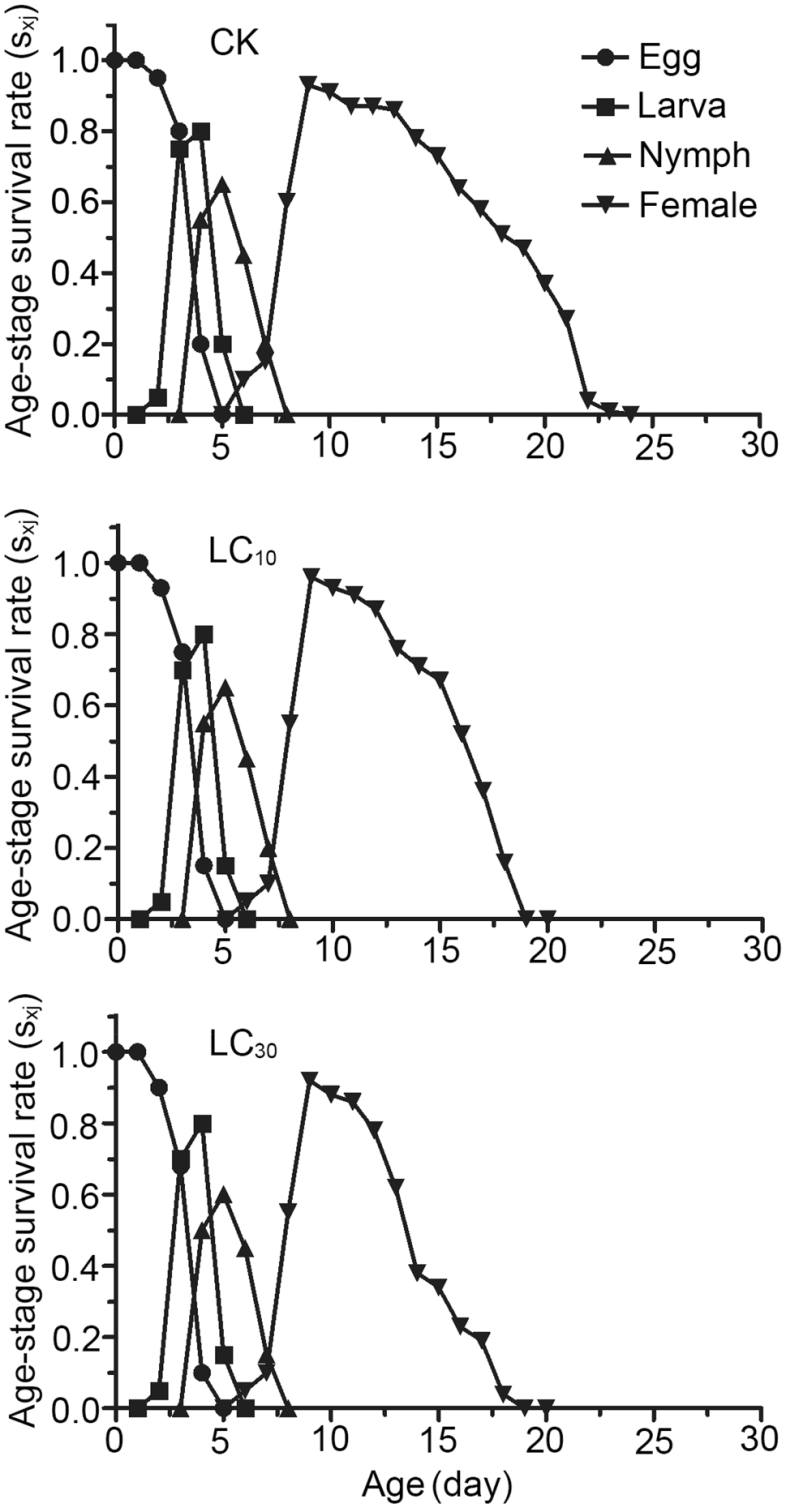
Age-stage specific s rate (*s*_*xj*_) of *Panonychus citri.* After exposure to low lethal concentrations of bifenazate.

As shown in Fig. 2, the probability of newborn eggs surviving to x age and developing to j stage is expressed by the survival rate of specific age stage (*s*_*xj*_), and the survival curves of different age stages overlap obviously; Compared with the control group, the survival rate of female adult mites in LC_10_ and LC_30_ treatment groups is relatively low.

**Figure 3:**
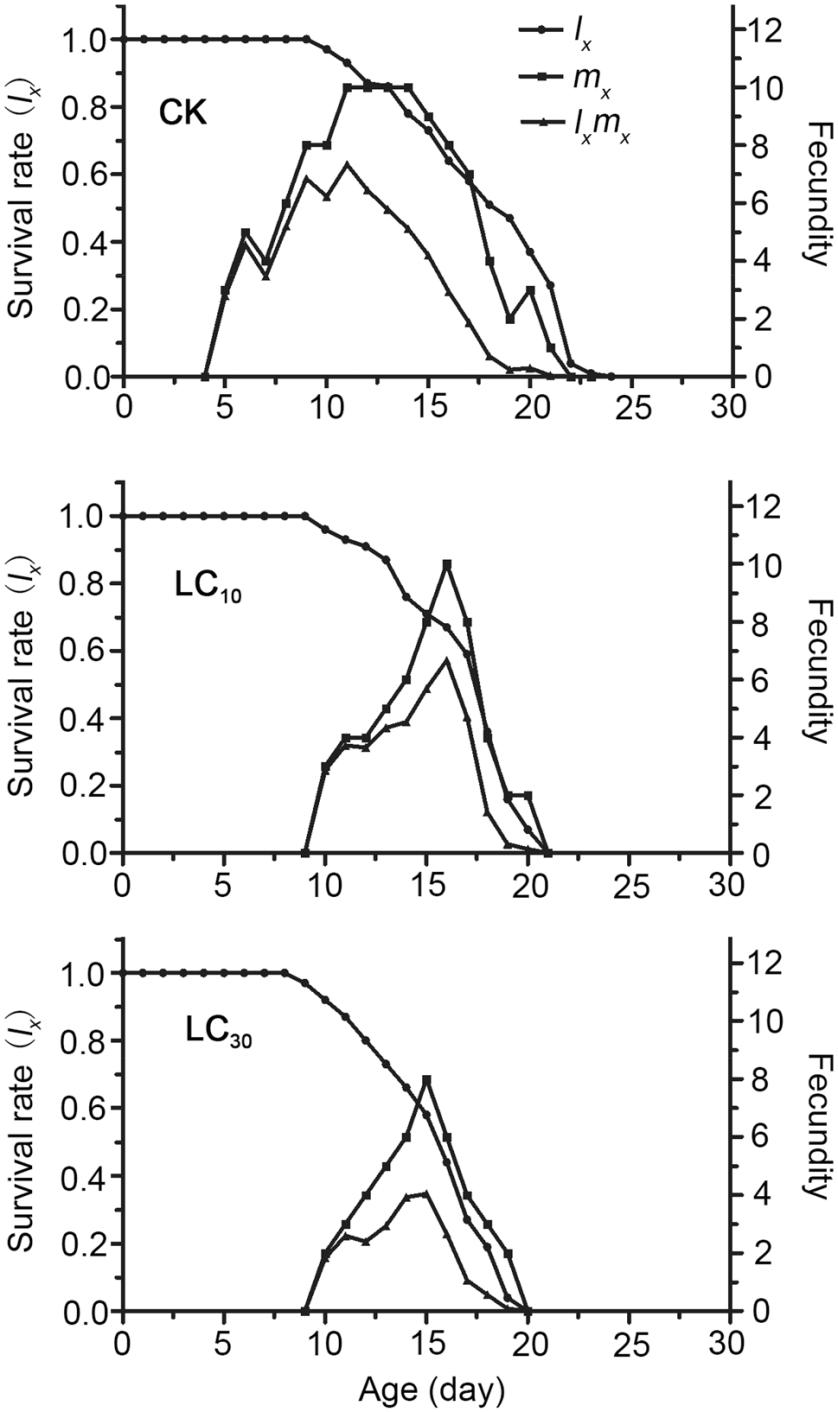
Age-specific survival rate (*l*_*x*_), age-specific feundity of the total population (*m*_*x*_), and age-specific maternity (*l*_*x*_*m*_*x*_) of *Panonychus citri*. After exposure to low lethal concentrations of bifenazate.

Age-specific survival rate (*l*_*x*_), age-specific fecundity (*m*_*x*_) and age-specific fecundity (*l*_*x*_*m*_*x*_) can be reflected in Fig. 3. *l*_*x*_ is the survival rate of newborn eggs to the age of x, *m*_*x*_ is the total fecundity of the population to the age of x, *l*_*x*_*m*_*x*_ is the result of the multiplication of *l*_*x*_ and *m*_*x*_; *l*_*x*_and *m*_*x*_ of LC_10_ and LC_30_ treatment are lower than the control, *l*_*x*_ and *m*_*x*_ show a downward trend with the increase of concentration.

### Low lethal concentration of bifenazate effects on protective enzyme activities of *P. citri*

**Figure 4:**
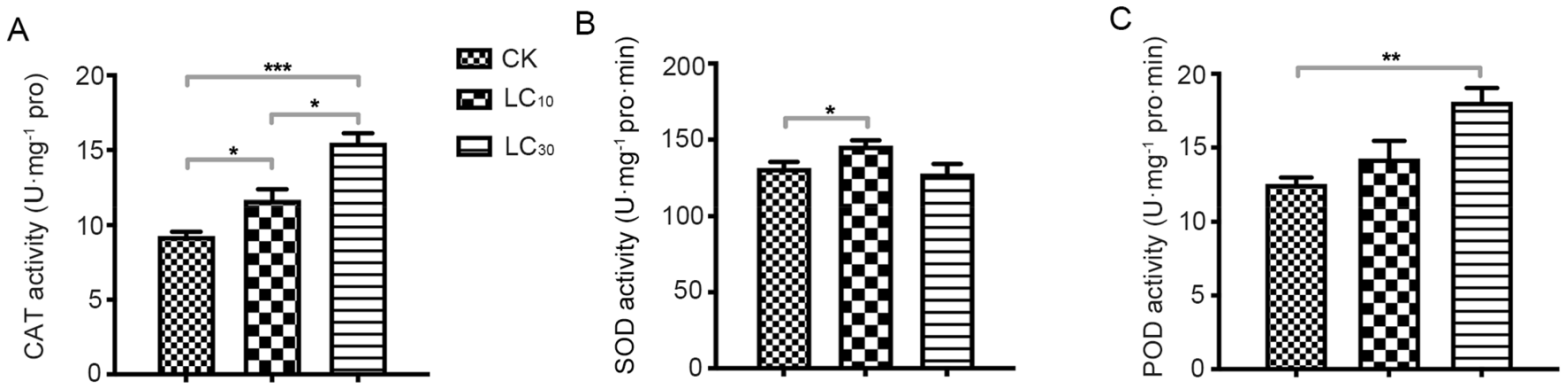
Effects of bifenazate stresses on antioxidant enzyme activity (U mg^−1^ protein min^−1^) of *Panonychus citri* (CK served as control group, data are means ± SE of three biological replications; different letters above each bar indicate statistically significant difference by ANOVA followed by the Duncan’s multiple range test) *p < 0.05, **p < 0.01, ***p < 0.001. (**A**) catalase (CAT), (**B**) superoxide dismutase (SOD), (**C**) peroxidase (POD).

The CAT、SOD 、POD activities of the third instar female adults *P.citri* treated with bifenazate was determined after 24 h. As shown in Fig. 4, the CAT activity of LC_10_ and LC_30_ treatment increased significantly compared with the control; the SOD activity of LC_10_ treatment increased, but the SOD activity of LC_30_ treatment group had no significant differences. Moreover, there was no significant difference in POD activity in the LC_10_ treated group compared with the control, However, POD activity was significantly higher in the LC_30_ treated group.

### Low lethal concentration of bifenazate effects on detoxifying enzyme activities of *P. citri*

**Figure 5:**
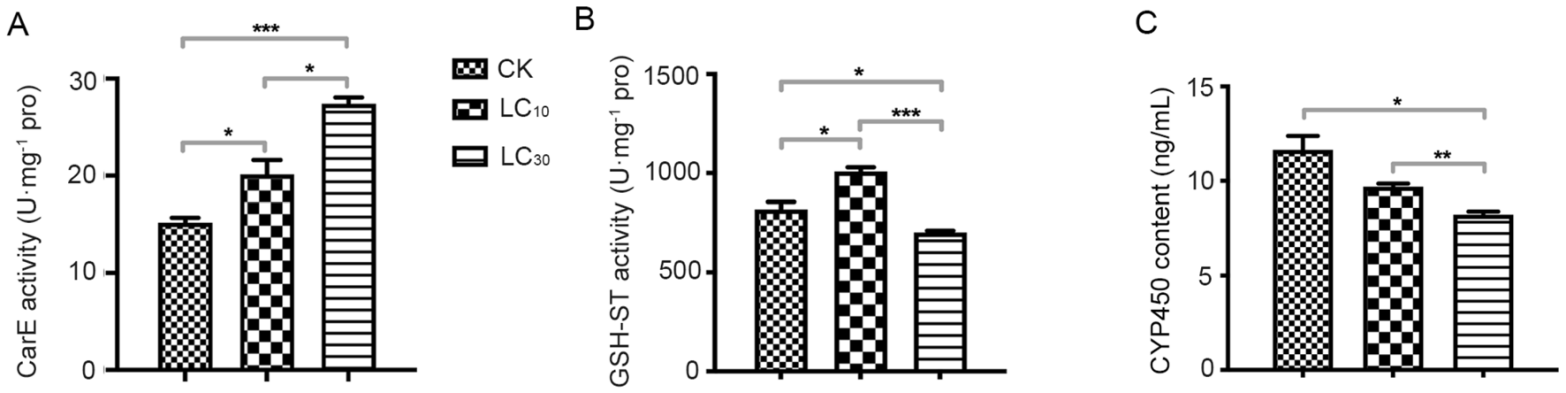
(**A,B**) Effects of bifenazate stresses on carboxylesterase (CarE), glutathione S-transferase (GSH-ST) activity (nmol mg^−1^ protein) of *Panonychus citri*; (**C**) effects of bifenazate stresses on Cytochrome P450 (CYP450) content of *P. citri*. (CK served as control group, data are means ± SE of three biological replications; different letters above each bar indicate statistically significant difference by ANOVA followed by the Duncan’s multiple range test). *p < 0.05, **p < 0.01, ***p < 0.001.

The activities of CarE, GSH-ST and CYP450 of the third instar female adults *P. citri* treated with bifenazate were determined 24 h later. As shown in Fig. 5, the activity of CarE in all treatment groups increased significantly compared with the control; the activity of GSH-ST in LC_10_ treatment increased, while in LC_30_ treatment decreased significantly; the activity of CYP450 in all treatment decreased compared with the control.

## Discussion

In pest control, it is important to assess the sublethal effects of insecticides on pest populations, and life table analysis is an important technique for effective assessment^35^. Fecundity, intrinsic rate of increase (*rm*), finite rate of increase (*λ*), and net reproductive rate (*R*_0_) are several key parameters for evaluating population growth, development and reproduction^36, 37^.

Our toxicity assay results showed that bifenazate is a potential insecticide for effective control of *P. citri*; However, insecticide has not only acute toxicity, but also the sublethal effects^38^. In the current study, the fecundity of *P. citri* was decreased in the LC_10_ or LC_30_ treated groups, and the same response was observed in pests treated with other insecticides^39^. For example, the low lethal concentrations (LC_20_, LC_30_) of chlorfenapyr inhibit *T. urticae* development and reproduction^40^. A low lethal concentration (LC_30_) of cyantraniliprole significantly inhibited fecundity in *Helicoverpa assulta*^11^. In contrast, many studies have shown that low concentrations of insecticides promote pest fecundity, for example, treatment with LC_10_ and LC_20_ of spinetoram reduces the time of development of spotted mites from eggs to adults and promotes their fecundity^20^. *Bradysia odoriphaga* reproduction was stimulated by chlorfenapyr^40^. In this study, we found that the low lethal concentration of bifenazate can effectively inhibit the increasing population of *P. citri*. This result showed that the logical application of insecticides is integral to *P. citri* management.

Insecticide stress has an impact on insect development, fecundity and population parameters^10^. In addition, they can also affect the activities of antioxidant enzymes and detoxification enzymes^41^. *Panonychus citri* exposure to different sublethal and low lethal concentrations of bifenazate (LC_10_, LC_30_) resulted in a significant increase in the activity of antioxidant enzymes, which was reduced near death (Fig. 4). Chemical stress causes the body to start its own defense system, produce a lot of reactive oxygen species, increase SOD activity and produce H_2_O_2_, which requires CAT and POD decomposition to achieve the relative balance of the body; when the concentration of chemical continues to rise, the self-defense ability will be weakened, resulting in the inhibition of protective enzyme activity^42, 43^; this is consistent with the previous studies on other pests using other insecticides^44^.

At the same time, it can also affect the activity of detoxification enzymes; CYP450, GSH-ST, and CarE are three key supergene families in insect detoxification metabolism^45^. GSH-ST is a family of enzymes that can catalyze the binding reaction of reduced glutathione (GSH) compounds, which has electrophilic properties and can catalyze the hydrolysis of esters, sulfates and amides. GSH-ST and CarE are the main enzymes involved in pesticide metabolism^46, 47^. Previous studies showed that the activities of CarE and GSH-ST were up-regulated in *Sogatella furcifera* treated with buprofezin at low lethal concentrations (LC_10_ and LC_25_) for 48 h, which increased with insecticide concentration rising^45^. In the present study, the activities of CarE and GSH-ST increased significantly after exposure to different low lethal concentrations (LC_10_, LC_30_) of bifenazate, and decreased when approaching death (Fig. 5). In addition, CYP450 is a multifunctional enzyme, which plays an important role in the formation of insecticide resistance^5^. In a variety of animal cells, it has been proved that a variety of insecticides can induce the production of reactive oxygen species (ROS), and then induce oxidative stress^48^. ROS attack, protein and lipid lead to oxidative damage, which destroys the integrity of enzyme structure and reduces enzyme activity^49^. At present, the results showed that compared with the control group, the CYP450 enzyme activity in the treatment group decreased significantly with the increase of the concentration of bifenazate. For example, when locusts were exposed to different sublethal doses of chlorpyrifos, the activity of CYP450 enzyme decreased with insecticide concentration increasing^50^. Therefore, these results provide a basis for further study on the molecular characteristics of antioxidant enzymes and detoxification enzymes of *P. citri*, and it is of great significance to understand the relationship between insecticides, antioxidant enzymes and detoxification enzymes.

In summary, bifenazate not only showed acute toxicity to *P. citri*, but also had sublethal effect. This study showed that sublethal and low lethal concentration of bifenazate could significantly affect the development duration and fecundity of *P. citri*. In addition, the activity of CYP450 in *P. citri* exposed to low lethal concentration was inhibited. Therefore, it is necessary to study bifenazate effects on CYP450 and reproductive related genes in *P. citri* at mRNA level in the future. It is an effective way to control the growth and development of *P. citri*.
